# Effect of Mo Dispersion on the Catalytic Properties and Stability of Mo–Fe Catalysts for the Partial Oxidation of Methanol

**DOI:** 10.3390/molecules25102410

**Published:** 2020-05-21

**Authors:** Shuai Zhang, Minghan Han

**Affiliations:** Department of Chemical Engineering, Tsinghua University, Beijing 100084, China; vstar_shuai@163.com

**Keywords:** Mo–Fe catalyst, Mo dispersion, stability, oxygen vacancies

## Abstract

Mo–Fe catalysts with different Mo dispersions were synthesized with fast (Cat-FS, 600 r·min^−1^) or slow stirring speed (Cat-SS, 30 r·min^−1^) by the coprecipitation method. Improving the stirring speed strengthened the mixing of the solution and increased the dispersion of particles in the catalyst, which exhibited favorable activity and selectivity. The byproduct (dimethyl ether (DME)) selectivity increased from 2.3% to 2.8% with Cat-SS, while it remained unchanged with Cat-FS in a stability test. The aggregation of particles and thin Mo-enriched surface layer decreased the catalyst surface area and slowed down the reoxidation of reduced active sites with Cat-SS, leaving more oxygen vacancies which promoted the formation of DME by the nonoxidative channel.

## 1. Introduction

Formaldehyde is an important industrial chemical [1,2]. The production of formaldehyde from methanol selective oxidation is based on a Mo–Fe catalyst [3,4], which offers a high formaldehyde yield and long catalyst lifetime. These advantages result in low methanol unit consumption and a high-quality product, i.e., formaldehyde aqueous solution. The lifetime of an industrial Mo–Fe catalyst is about 8 to 18 months; it is recognized that the deactivation of Mo–Fe catalysts is due to Mo loss and phase separation into MoO_3_ and Fe_2_O_3_, which decreases activity and selectivity [3,5,6]. In industrial use, the Mo/Fe mole ratio of the catalyst is usually above 2.0, rather than the stoichiometric ratio of iron molybdate to compensate for Mo loss during the reaction [7,8]. Therefore, the catalyst consists not only of Fe_2_(MoO_4_)_3_, but also of MoO_3_. However, MoO_3_ should be prevented from forming plate-like structures in the preparation process due to its low activity and selectivity [9,10,11,12].

The reaction for methanol selective oxidation to formaldehyde (Equation (1)) and the main byproduct (DME) are

CH_3_OH + 0.5 O_2_ = CH_2_O + H_2_O (1)

2CH_3_OH = CH_3_OCH_3_ + H_2_O (2)

Factors like Mo and Fe precursors, concentration of initial solutions, temperature, pH during precipitation, stirring speed, aging of the precipitate and calcination conditions are important [13,14,15,16]. Many researchers have focused on the effect of the structure on catalytic activity rather than its stability. In this work, catalysts with different Mo dispersions, that is, different contents of amorphous Mo-rich surface layers or plate-like MoO_3_ with identical Mo/Fe mole ratios, were synthesized by controlling the stirring speed during catalyst preparation. The stirring speed affected the dispersion of iron molybdate particles in the catalysts. These factors jointly influenced the activity, selectivity and stability of the catalysts.

Most authors agree that the mechanism over Mo–Fe catalysts is a redox mechanism involving the reduction of the active site of the catalyst during the reaction and reoxidation of the reduced catalyst, that is, a Mars-van Krevelen mechanism [9,17]. In this work, the reoxidation of the reduced catalyst is discussed based on a characterization of the behavior of the oxygen species, such as oxygen vacancy and lattice oxygen, of fresh and used catalysts, to understand the connection between the structure and catalytic performance.

## 2. Experimental

### 2.1. Materials

Ammonium heptamolybdate tetrahydrate (AHM), iron nitrate nonahydrate and methanol were purchased from Sinopharm Chemical Reagent Ltd. Corporation, Shanghai, China. Nitric acid and ammonia, with mass fractions of 65–68% and 25–28%, respectively, were obtained from Beijing Chemical Works. 

### 2.2. Preparation of Catalysts

The catalysts were prepared by the coprecipitation method with different stirring speeds. The speed for Cat-SS was 30 r·min^−1^, and for Cat-FS was 600 r·min^−1^. AHM and iron nitrate were dissolved in deionized water at concentrations of 0.29 mol·L^−1^ and 0.94 mol·L^−1^, respectively, and a Mo/Fe mole ratio of 2.60. The ferric nitrate solution was added dropwise to the AHM solution over 30 min at 60 °C. The pH value was tested by phs-3C pH meter, and maintained at pH = 2.0 by adding ammonia. The precipitate was collected by suction filtration, washed, dried in an oven at 60 °C for 12 h, and then calcined in a muffle furnace at 500 °C for 24 h.

### 2.3. Catalytic Test and Characterization

The reaction was carried out in a microreactor (6 mm i.d. × 70 cm). The calcined catalyst was ground into 100–300 mesh powder and 0.50 g was placed in the middle of the microreactor. The flow rate of air was 106.6 mL·min^−1^, and methanol was pumped in at a flow rate of 0.011 mL·min^−1^ with methanol mole ratios of N_2_: O_2_ = 1: 13.2: 12.9. An activity test was carried out between 250 °C and 300 °C. A stability test was carried out at 300 °C for 150 h. The product was sampled online at 120 °C and analyzed using a gas chromatograph (GC, 9790IIT-2, FULI, ZheJiang, China) equipped with a TCD detector and a packed column (Poropak N, 3 mm × 5 m, Hichina Zhicheng Technology Ltd., Gansu, China).

In addition to the main product, i.e., formaldehyde, DME was also detected as a byproduct. The relative contents of both products were determined by the normalization method. The conversion of methanol and the selectivity of the products were calculated as:

Methanol conversion (%) = moles of converted methanol/moles of methanol feedstock × 100%(3)

Products selectivity (%) = moles of products/moles of converted methanol × 100%(4)

The crystal structure was identified by Powder X-ray diffraction (XRD) using an X-ray powder diffractometer (Bruker-AXS D8 Advance, Karlsruhe, Germany) with a Cu K_α_ radiation source with an operating speed of 10 degree·min^−1^. A scanning electron microscope (SEM, JEM 7401F, JEOL, Tokyo, Japan) was used to characterize the morphology of the catalysts. A transmission electron microscope (TEM, JEM-2010, JEOL, Tokyo, Japan) was used to examine the difference between the catalyst bulk and interface. High-angle annular dark-field scanning TEM (HAADF-STEM) was performed using a JEOL ARM200F microscope (JEOL, Tokyo, Japan) with a STEM aberration corrector operating at 100 kV. Energy-dispersive X-ray spectroscopy (EDS) was used to analyze the component in selected areas of the catalysts. An inductively coupled plasma optical emission spectrometer (ICP-OES, Spectro Arcos FHX22, Kleve, Germany) was used to determine the Mo/Fe mole ratio of the whole catalyst. The specific surface area and pore size distribution of the catalysts were determined by N_2_ adsorption using a Quadrasorb-S1 instrument (Quantachrome, Florida, USA) and the Brunauer-Emmett-Teller (BET) and the Barrett-Joyner-Halenda (BJH) method, respectively. X-ray photoelectron spectroscopy (XPS) analysis was performed on a Thermal Scientific ESCALAB 250Xi instrument using an Al K_α_ X-ray source.

The O_2_-TPD of the catalysts was measured by O_2_ temperature programmed desorption (TPD, Quantachrome Instruments, Chembet PULSAR). The sample was flushed with He at 300 °C for 30 min, and then cooled to 30 °C and kept under flowing 5% O_2_/He for 30 min. Physically adsorbed O_2_ was removed by flushing with He at 100 °C for 30 min. Then, chemically adsorbed O_2_ on the catalyst was measured by heating from 100 to 800 °C at a rate of 10 °C·min^−1^. The H_2_-TPR profiles of the catalysts were determined by temperature programmed reduction (TPR, Quantachrome Instruments, Chembet PULSAR). The samples were flushed with He at 300 °C for 30 min and then cooled to 30 °C. 10% H_2_/He was switched on and the flow rate through the reactor was controlled at 100 mL/min. The temperature was increased at a rate of 10 °C·min^−1^ from 30 to 1000 °C. The H_2_ consumption (TCD signal) was recorded by a PC.

## 3. Results and Discussion

### 3.1. Characterization of the Catalysts 

**Figure 1:**
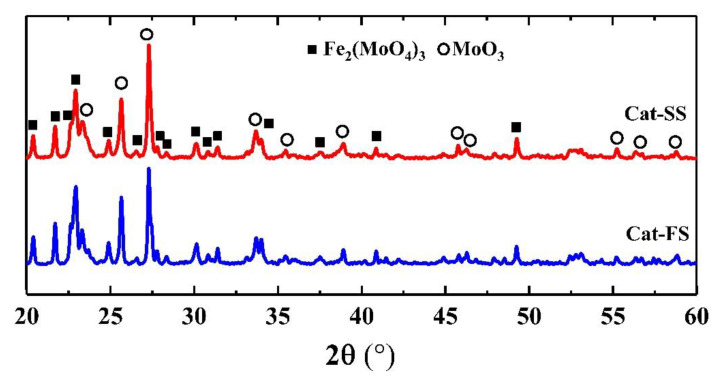
X-ray diffraction patterns of Cat-SS and Cat-FS.

Figure 1 shows the XRD patterns of two catalysts, where 2θ = 20.4°, 21.7°, 22.9°, 24.9°, 26.6°, 30.2°, 40.9° and 49.5° belonged to the diffraction peak of Fe_2_(MoO_4_)_3_, and 2θ = 23.1°, 25.7°, 27.3°, 34.1°, 38.9° and 55.1° were assigned to MoO_3_. It was shown that the catalyst consisted of Fe_2_(MoO_4_)_3_ and MoO_3_.

**Figure 2:**
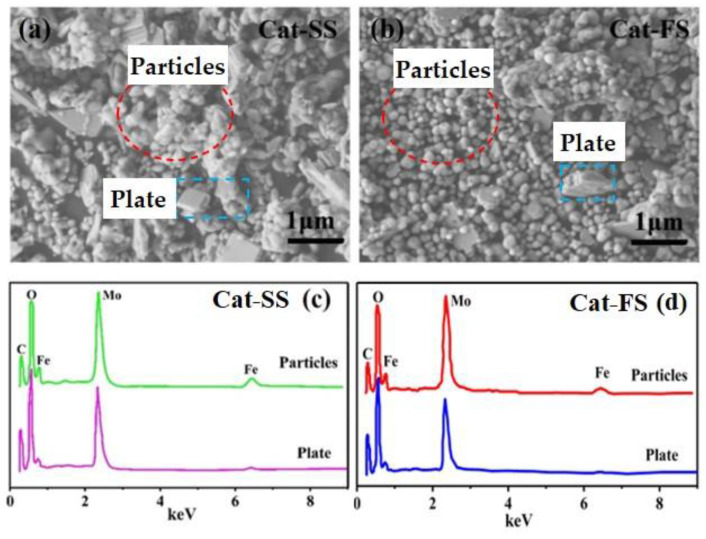
SEM images of Cat-SS (**a**) and Cat-FS (**b**), (**c**) EDS data of the particles and plates marked in (**a**), (**d**) EDS data of the particles and plates marked in (**b**).

Figure 2a,b show SEM images of the two catalysts. It can be clearly seen that the catalysts comprised particle-and plate-like structures. Cat-SS has more plate-like structures and showed an obvious agglomeration of particles. However, Cat-FS has few plate-like structures and the particles were dispersed uniformly.

Figure 2c,d display the EDS spectra of particles and plates marked in the SEM images of Figure 2a,b, respectively. The Mo/Fe mole ratio of particles and plates are shown in Table 1. The mole ratio of particles was between 1.6 and 1.8, while the mole ratio of the plates ranged from 4.8 to 5.5. The mole ratio of the particles was lower than the plate-like structure. It was concluded that the particles and plates could be assigned to Fe_2_(MoO_4_)_3_ and MoO_3_, respectively. Due to that the particles stuck to the plates, the mole ratio of MoO_3_ was not theoretical infinite. 

The Mo/Fe mole ratio of the whole Cat-FS was identical with Cat-SS, which was accurately analyzed by ICP-OES, as shown in Table 1. However, the EDS data, as shown in Table 1, illustrates that the Mo/Fe mole ratio of the Fe_2_(MoO_4_)_3_ particles of Cat-FS was higher than those of Cat-SS, which implied that there was more Mo on these particles. The XPS results, as shown in Table 1, show that the Mo/Fe mole ratio of Cat-FS was slightly higher than that of Cat-SS, which also implies that more Mo enriched on the surface layer of particles in Cat-FS than in Cat-SS, because the XPS method focused more on the surface layer.

**Figure 3:**
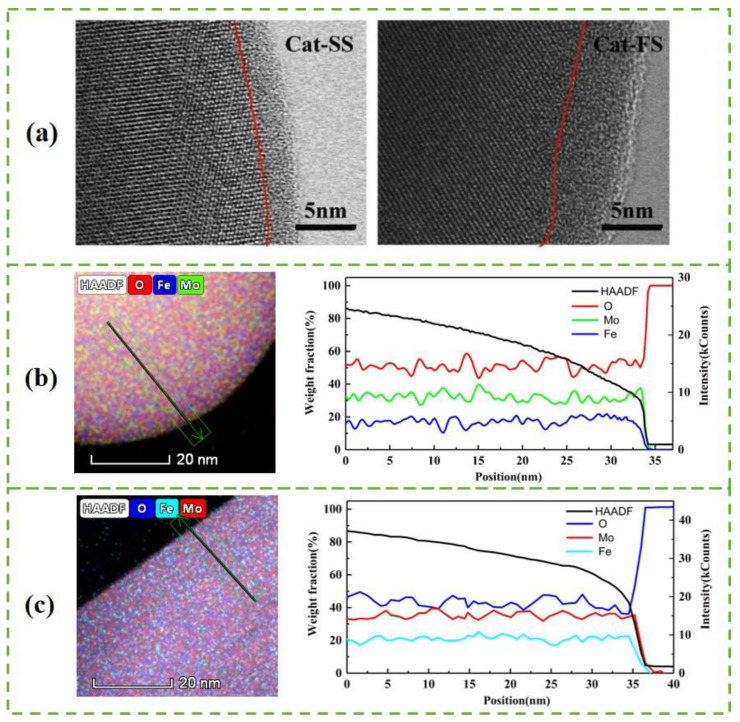
(**a**) STEM images of particles with Cat-SS and Cat-FS, HAADF-STEM image and STEM-EDS line scan of particles in Cat-FS (**b**) and Cat-SS (**c**) showing the Mo, Fe and O distribution along the arrow marked in the HAADF-STEM image.

Figure 3a shows the STEM images of particles with two catalysts. A 3–4-nm-thick amorphous surface layer was observed on the bulk Fe_2_(MoO_4_)_3_ crystallites on Cat-SS. Cat-FS has a thicker amorphous layer than Cat-SS. Figure 3b,c show a STEM-EDS line scan, giving the amount of Mo, Fe and O in the area across the particle marked in the HAADF-STEM image of Cat-SS and Cat-FS, respectively. It is shown that there was no gradient in the Fe or Mo content on the bulk Fe_2_(MoO_4_)_3_, but that the amount of Fe decreased more than that of Mo on the surface layer. This illustrates that the amorphous layer was enriched in Mo and depleted in Fe compared with the bulk composition [14], that is, there was a thicker Mo-enriched surface layer on the particles of Cat-FS. The strengthened mixing of the solution at a fast stirring speed caused the excess Mo species to be better dispersed on Cat-FS than on Cat-SS, and increased the thickness of the amorphous layer on Cat-FS. More uniformly dispersed particles and more polyporous amorphous layers in the Cat-FS may have increased the surface area of the catalyst, as shown in Table 1.

### 3.2. Activity and Stability of the Catalyst

**Figure 4:**
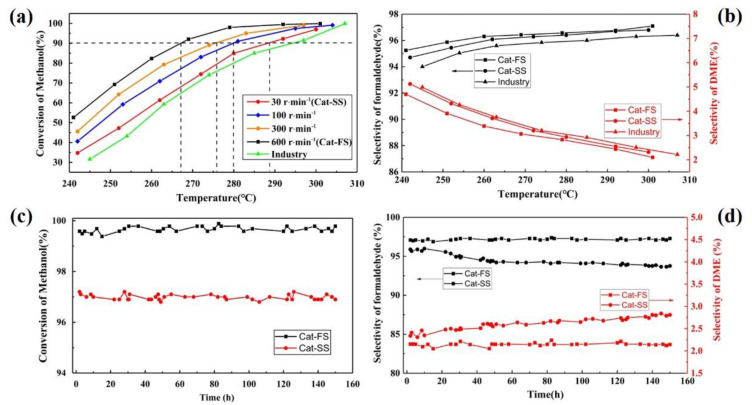
(**a**) Activity and (**b**) selectivity of catalysts. Stability of catalysts: (**c**) conversion and (**d**) selectivity of the catalyst.

Figure 4a shows the activity of catalysts synthesized at different stirring speeds. When the methanol conversion reached 90%, the reaction temperature increased along with the stirring speed, which implies that the activity of the catalyst increases as the stirring speed increases. Figure 4b shows the selectivity of Cat-FS and Cat-SS. The Cat-FS had higher formaldehyde selectivity than both Cat-SS and the catalyst used in industry. Improving the stirring speed strengthened the mixing of the solution and increased the dispersion of particles in the catalyst. The fast stirring speed used in the catalyst preparation resulted in a thicker Mo-enriched amorphous surface layer and higher specific surface area, which may exhibit favorable activity and selectivity.

The stability of the catalysts was also tested at 300 °C for 150 h, as shown in Figure 4c,d. With Cat-FS, the methanol conversion and formaldehyde selectivity remained unchanged for 150 h. However, although methanol conversion of Cat-SS was unchanged, the selectivity of the formaldehyde decreased from 96.7% to 96.2%, and that of DME increased from 2.3% to 2.8%. These results illustrated that Cat-SS had not only poor activity and selectivity, but also poor stability. 

### 3.3. Analysis of Fresh and Used Catalysts

**Figure 5:**
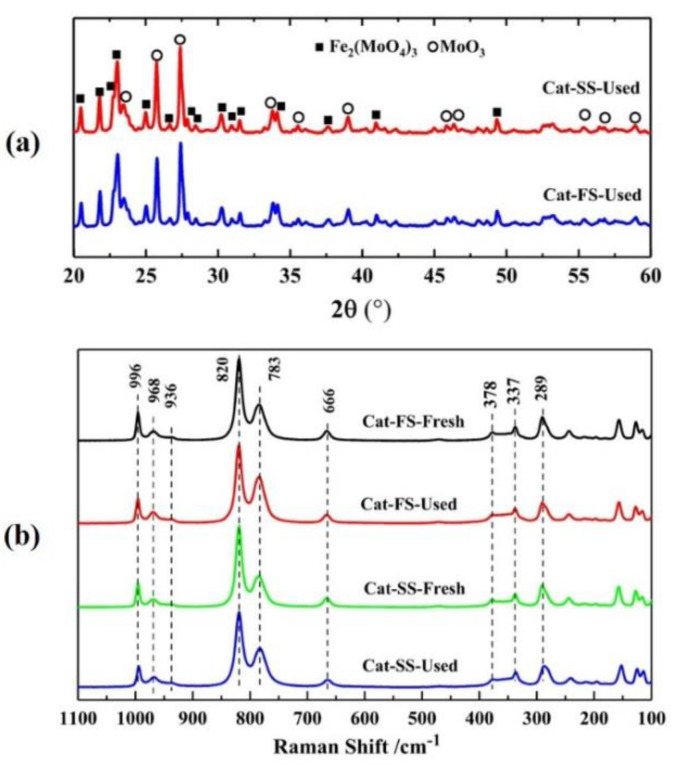
(**a**) X-ray diffraction patterns of Cat-SS and Cat-FS; (**b**) Raman spectra of fresh and used catalysts.

Figure 5a shows the XRD patterns of catalysts after stability testing; the XRD patterns are almost identical with those of fresh catalysts. All the diffraction peaks were assigned to Fe_2_(MoO_4_)_3_ and MoO_3_, implying that the structure of two catalysts had not changed. Figure 5a illustrates the Raman spectra of fresh and used catalysts, where the lines at 968, 936, 783, 352 and 337 cm^−1^ belong to Fe_2_(MoO_4_)_3_, and those a of 996, 820, 669, 337 and 289 cm^−1^ to MoO_3_. The used catalyst was similar to the fresh catalyst, implying that no new phase composition was generated after stability testing. Moreover, the Mo/Fe mole ratio of the used catalysts was not significantly lower than that of the fresh catalysts analyzed by ICP and XPS, as shown in Table 1. These results exclude the effect of changes in the Mo/Fe mole ratio and the structure of catalysts on the DEM selectivity of Cat-SS in the stability testing.

**Figure 6:**
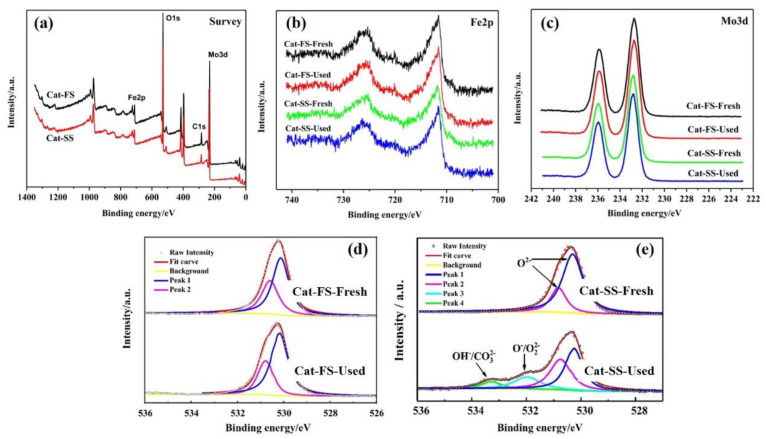
XPS profiles of fresh and used catalysts: (**a**) Survey, (**b**) Fe2p, (**c**) Mo3d, (**d**) O1s of Cat-FS and (**e**) O1s of Cat-SS.

Figure 6a shows the XPS survey of Cat-FS and Cat-SS, which implied that both catalysts were composed of three elements: Mo, Fe and O. Figure 6b,c show the Fe2p and Mo3d XPS results of fresh and used catalysts, respectively. The XPS spectra exhibited similar peaks, showing that Mo and Fe remained unchanged after the stability test lasting 150 h. Figure 6e shows the O1s XPS results of the fresh and used Cat-SS. The peaks at 530.2 and 530.9 eV were attributed to oxygen ions in the crystal lattice (O^2−^). The second peak at 532.0 eV was assigned to adsorbed oxygen species (O^−^/O_2_^2−^). The third peak with the highest binding energy at 533.2 eV was assigned to hydroxyl and carbonate species (OH^−^/CO_3_^2−^) [18]. The appearance of the second and third peaks suggested that the reaction was consuming the oxygen in the Cat-SS during the long-lasting testing, which generated oxygen vacancies [19] due to slow replenishment by gas phase O_2_ in air. However, the XPS profile of the used Cat-FS was quite the same as that of fresh Cat-FS, as shown in Figure 6d, suggesting that consumed lattice oxygen on Cat-FS was replenished by gas phase O_2_ over time. Determining the behavior of O species in a long-duration test is important to understanding the mechanism of the reaction that resulted in poorer formaldehyde selectivity.

**Figure 7:**
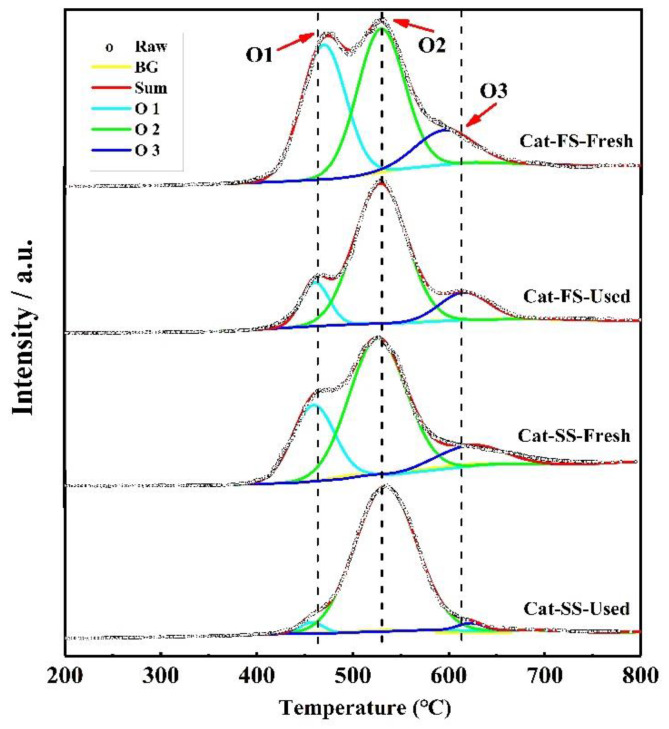
O_2_-TPD results of fresh and used Cat-FS and Cat-SS.

Figure 7 shows the O_2_-TPD results of fresh and used catalysts; the three peaks were attributed to weak chemisorbed oxygen species (O1), strong chemisorbed oxygen species (O2) and lattice oxygen species (O3) [20,21,22]. With the used catalysts, the intensity of the O1 peak of Cat-FS was higher than that of Cat-SS, indicating that it had more adsorbed oxygen species. The intensity of the O1 and O3 peaks of Cat-FS were little changed after the long-duration test, but, in contrast, these nearly disappeared for Cat-SS after the long-duration test; this was accompanied by strong intensity of the O2 peak. The strongly chemisorbed oxygen species of the O2 peak was associated with oxygen vacancies [23,24], which are harmful to the selectivity to formaldehyde. 

**Figure 8:**
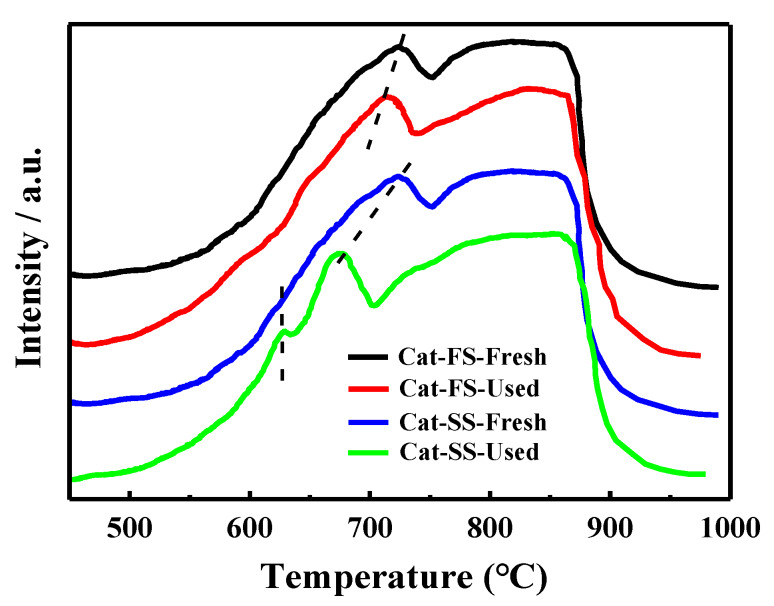
H_2_-TPR results of fresh and used Cat-FS and Cat-SS.

Figure 8 shows the H_2_-TPR results of the fresh and used catalysts. MoO_3_ and Fe_2_(MoO_4_)_3_ were reduced to MoO_2_ and FeMoO_4_, respectively, in the temperature range from 550 to 750 °C, and the reduced species were further reduced when the temperature exceeded 750 °C [25]. The reduction profile of fresh Cat-FS was similar to that of fresh Cat-SS. However, the reduction temperature of the used Cat-FS was slightly lower than that of the fresh catalyst, indicating that a small amount of active oxygen species was produced during the long-duration reaction. It was obvious that the reduction temperature of the used Cat-SS was much lower than the fresh catalyst, and a small peak appeared at 630 °C, which demonstrated that many more active oxygen species were generated during the reaction. 

**Figure 9:**
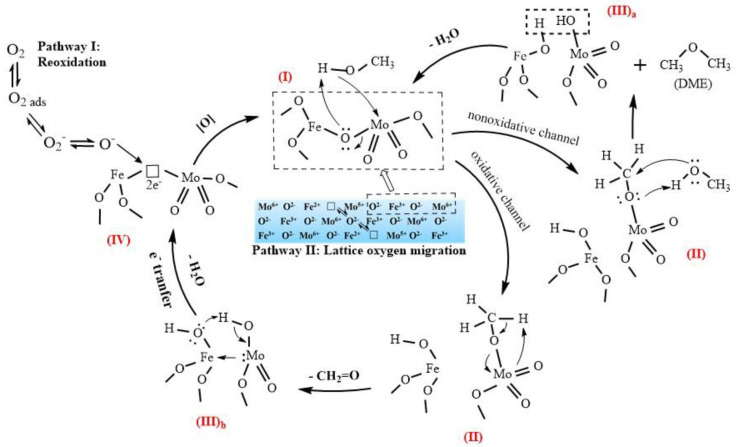
Suggested mechanism of the selective oxidation of methanol to formaldehyde over Mo–Fe catalysts.

Figure 9 shows the suggested mechanism of the selective oxidation of methanol to formaldehyde and DME over a Mo–Fe catalyst, denoted as species (I). This reaction mechanism is a Mars-van Krevelen mechanism, which can be broken down into three steps [26]: (1) the dissociative adsorption of methanol, (2) the oxidation and desorption of products, leaving an oxygen vacancy on the surface of the catalyst, and (3) the replenishment of the oxygen vacancy.

The initial step is the dissociative chemical adsorption of methanol to form surface methoxy (II) [27]. The methoxy intermediate can react via either an oxidative or nonoxidative channel. The nonoxidative channel involves the reaction with physically adsorbed methanol molecules to produce DME and the (III)_a_ intermediate [28]. The original structure (I) is restored by the removal of water from the (III)_a_ intermediate.

The formation of formaldehyde occurs due to the oxidative channel of the second step. Hydrogen is abstracted from adsorbed methoxy by a terminal oxygen in Mo=O to produce a transient adsorbed CH_2_=O_(a)_ intermediate that desorbs from the active site, which forms an intermediate with two hydroxyl (III)_b_ [29]. After the removal of the water, there is an oxygen vacancy with two electrons on the surface (IV).

The catalytic reaction needs the participation of lattice oxygen, which leads to the formation of an oxygen vacancy, and the active center on the surface is left in a reduced state. Before it can be used again, the active center must be reoxidized. The reoxidation can be carried out either by the incorporation of oxygen from the gas phase (Pathway I) or the diffusion of lattice oxygen ions from the bulk and the electrons from the surface to the bulk (Pathway II) [22,30]. If the active center cannot be regenerated in time, it will remain in a reduced state. In this state, it will produce DME as a byproduct through the nonoxidative channel rather than formaldehyde through the oxidative channel, which needs to abstract the hydrogen in the methoxy by a terminal oxygen. This is because the methoxy on an oxidized site has a relatively stronger C–H bond than that on a reduced site [29].

The effect of the Mo-enriched layer on the catalytic properties is unknown and will be studied in the future, but we can explain the increase in DME selectivity during the lifetime test with Cat-SS now. It probably adsorbed more oxygen from the gas phase when the Mo species formed a Mo-enriched surface layer rather than forming plate-like MoO_3_, which led to a faster rate of O_2_ transfer from air into lattice oxygen with Cat-FS. However, it is inferred that less O_2_ transfer from air into lattice oxygen caused the reduced active center of Cat-SS, generating more nonoxidative channel DME during the long-duration reaction. It is necessary for the oxygen vacancy to be reoxidized in time for the activity and selectivity to remain constant in the long term. Raun [31] also found that the Mo structure could influence the stability of Mo–Fe catalysts. A lower rate of catalyst deactivation was observed for the large h-MoO_3_ compared with α-MoO_3_.

In summary, the Cat-SS agglomerated particles with a thinner Mo-enriched surface layer and more plate-like MoO_3_ than Cat-FS, which also decreased the surface area of the Cat-SS. These factors reduced the activity and selectivity, as well as the stability of the catalyst. A ow Cat-SS surface area slowed down the adsorption of gas phase oxygen, which caused Cat-SS to consume lattice oxygen and produced oxygen vacancies during the oxidation-reduction cycle. Therefore, in order to enhance the activity and stability of the catalyst, the stirring speed during the preparation should be maximal to avoid forming aggregated particle structures and increase the dispersion of Mo species to enable the formation of a thick Mo-enriched surface layer, rather than MoO_3_ plates.

## 4. Conclusions

Mo–Fe catalysts were prepared by the coprecipitation method with a slow or fast stirring speed, and their catalytic performance were studied. The catalyst consisted of particle-like Fe_2_(MoO_4_)_3_ and plate-like MoO_3_. Fast stirring during catalyst preparation enhanced the dispersion of the particles and formed a thicker Mo-enriched surface layer on the particles. However, the catalyst prepared at a slow stirring speed aggregated severely and had a thinner Mo-enriched surface layer on the particles, which decreased the surface area and led to poor activity, selectivity and stability. This resulted in slower reoxidation of the reduced active sites and formed oxygen vacancies during the reaction, which promoted the formation of DME by the nonoxidative channel as a byproduct.

## Figures and Tables

**Table 1:** The Mo/Fe mole ratio and surface areas of catalysts.

	Mo/Fe Mole Ratio-EDS		Mo/Fe Mole Ratio		Surface Area/m^2^·g^−1^
	Plate	Particles	ICP	XPS	
Cat-SS	5.5	1.6	2.50/2.50	2.49/2.51	15.6
Cat-FS	4.8	1.8	2.50/2.51	2.54/2.53	18.8

Note: the ICP and XPS column have two Mo/Fe mole ratios. The left data refer to the fresh catalyst and the right data to the used catalyst after the stability test in Figure 4.
